# Environmental risks from artificial nighttime lighting widespread and increasing across Europe

**DOI:** 10.1126/sciadv.abl6891

**Published:** 2022-09-14

**Authors:** Alejandro Sánchez de Miguel, Jonathan Bennie, Emma Rosenfeld, Simon Dzurjak, Kevin J. Gaston

**Affiliations:** ^1^ Environment and Sustainability Institute, University of Exeter, Penryn, Cornwall TR10 9FE, UK.; ^2^ Departamento de Física de la Tierra y Astrofísica, Instituto de Física de Particulas y del Cosmos (IPARCOS), Universidad Complutense, Madrid, Spain.; ^3^ Centre for Geography and Environmental Science, University of Exeter, Penryn, Cornwall TR10 9FE, UK.

## Abstract

The nighttime environment of much of Earth is being changed rapidly by the introduction of artificial lighting. While data on spatial and temporal variation in the intensity of artificial lighting have been available at a regional and global scale, data on variation in its spectral composition have only been collected for a few locations, preventing variation in associated environmental and human health risks from being mapped. Here, we use imagery obtained using digital cameras by astronauts on the International Space Station to map variation in the spectral composition of lighting across Europe for 2012–2013 and 2014–2020. These show a regionally widespread spectral shift, from that associated principally with high-pressure sodium lighting to that associated with broad white light-emitting diodes and with greater blue emissions. Reexpressing the color maps in terms of spectral indicators of environmental pressures, we find that this trend is widely increasing the risk of harmful effects to ecosystems.

## Teaser

Environmental risks of artificial nighttime lighting are increasing because of shifting spectra.

## INTRODUCTION

The natural nighttime environment has been eroded over large areas of Earth through the introduction of artificial lighting (*1*, *2*). This loss has particularly accelerated over the past 100 years, with the widespread introduction of electric-powered streetlights and other outdoor sources (*3*). Evidence for the geographic occurrence and extent of these changes to the nighttime light environment (exemplified by the now familiar maps of the world at night) (*1*, *2*, *4*) and their coincidence with and, thus, potential impacts on important biological features (e.g., ecosystems, vegetation types, species distributions, biodiversity hot spots, key biodiversity areas, and protected areas for biological conservation) (*5*–*9*) has focused almost exclusively on measurements of the intensity of light emissions and principally those obtained using satellite-borne sensors. While this has led to many important insights, it has, from a biological perspective, been problematic for three main and linked reasons. First, the key satellite sensors have been panchromatic, providing no information on the spectral composition of artificial light at night (ALAN), and while the bandwidths have been broad, they have also been insensitive to the blue part of the visible spectrum (380 to 450 nm); the Defense Meteorological Program/Operational Line-Scan System (DMSP/OLS) was sensitive from 450 to 1000 nm, and the Suomi-National Polar-Orbiting Partnership/Visible and Infrared Imaging Radiometer Suite–Day/Night Band (SNPP/VIIRS-DNB) and NOAA20 VIIRS-DNB are sensitive from 480 to 920 nm (*10*). Second, ALAN has been found to affect a wide diversity of biological phenomena, from individual physiology and behavior (including that of humans) to community structure and ecosystem function (*11*, *12*), with these responses almost invariably being dependent on the spectrum of emissions and some of the most important (e.g., melatonin suppression) being particularly sensitive to those at blue wavelengths (*13*–*15*). Third, much of the world’s outdoor lighting stock has been transitioning from narrow spectrum (e.g., low-pressure sodium) to “broad white” spectrum [using light-emitting diode (LED)] lamps, particularly resulting in increases in emissions in the blue part of the visible spectrum (*16*, *17*). Together, these issues mean that satellite-borne imagery may provide a poor or limited quantitative indicator of the exposure to environmental risks associated with ALAN, as shifts toward potentially harmful lower-wavelength emissions are masked by the low sensitivity of the sensors at these wavelengths.

To map the risks that ALAN poses to key biological processes at regional or continental scales in a fashion akin to that done for other anthropogenic pressures, there is a need for remotely sensed data on the spatial and temporal variation in the spectral composition of ALAN. At present, the most important sources of such data are the color photographs of Earth taken using digital single-lens reflex (DSLR) cameras by astronauts onboard the International Space Station (ISS) (*18*). Although not obtained systematically, a large number of these images have been taken during the nighttime (an estimated 1.25 million) over an extended period (2003 to the present). To date, this resource has only been exploited for quite localized studies [e.g., (*19*, *20*)], and problems of locating, compiling, and appropriately calibrating required images have only recently been resolved (*18*).

**Fig. 1.**
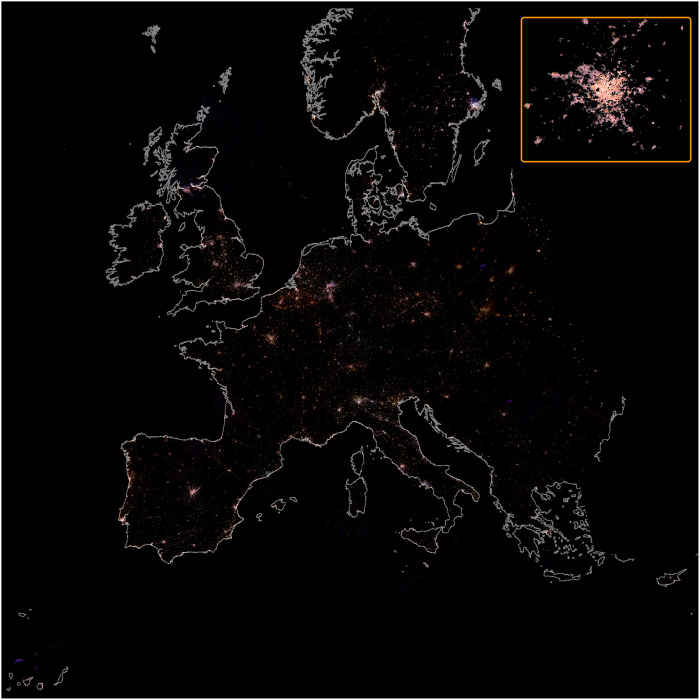
Color map of Europe at night for 2014–2020. Map created from images obtained from the ISS using Nikon D3, D3S, D4, and D5 DSLR cameras. These images have been calibrated and spatially mosaicked. The insert shows Paris as example of detail.

Here, we report the first composite nighttime color maps, produced using imagery obtained from the ISS that has been passed through a pipeline of processing and calibration (see Materials and Methods) for an extensive region, that of Europe. These maps were created for the periods 2012–2013 and 2014–2020 (Fig. 1), before and after the marked spread of LED streetlight technology and the associated changes in spectra of emissions were reported to take place. We used a synthetic photometry approach to estimate the spectral characteristics of sources from color ratios in the photographic images. We then used previously documented relationships between color ratios and environmental responses to map across a large area the environmental risks posed by these changing emission patterns.

## RESULTS

### Shifting spectra

**Fig. 2.**
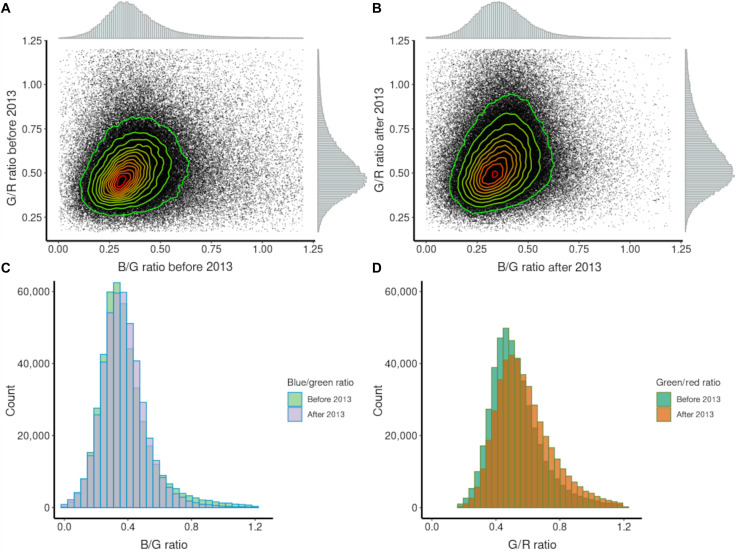
Temporal change in color ratios. Relationships (with associated frequency distributions) between B/G and G/R color ratios of nighttime light emissions across Europe at 500-m resolution for (**A**) 2012–2013 and (**B**) 2014–2020. Contours show density of points with red showing the highest density and green showing the lowest density. Histograms of the frequencies of 500-m pixels with different (**C**) B/G and (**D**) G/R ratios for 2012–2013 and 2014–2020.

**Fig. 3.**
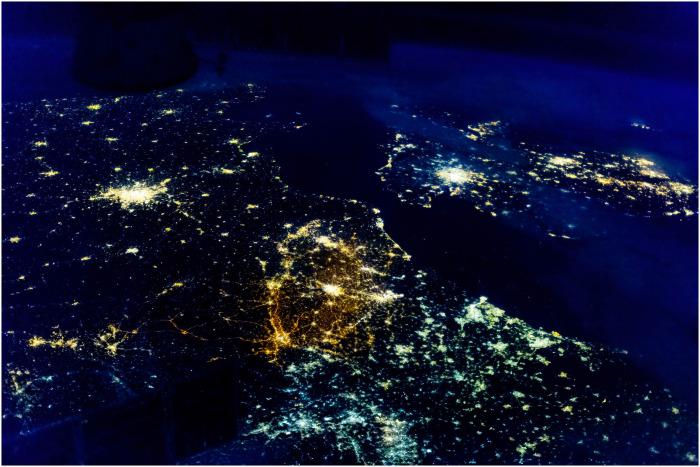
Belgium and surrounding countries at night. Color-enhanced ISS image (iss038e014887, 10-12-2013). Belgium in the middle with orange color, characteristic of use of low-pressure sodium lights and with roads lit. The Netherlands, France, and the United Kingdom, at the right, left, and top, respectively, with yellowish color from high-pressure sodium lights. Germany at the bottom, with blueish color from fluorescent and mercury vapor lights. Figure S3 provides a reprojected version of this image, with country boundaries.

Expressing nighttime artificial light emissions as blue/green (B/G) and green/red (G/R) ratios reveals key features of the spatial and temporal variation in their spectrum. In general, pixels with lower B/G and G/R ratios appear more orange in the images (often because of sodium lighting), while pixels with higher B/G and G/R ratios appear whiter. In 2012–2013, these ratios varied quite substantially across Europe (Fig. 2 and fig. S1), with differences often following national boundaries, Belgium and the Republic of Ireland, for example, having quite orange light, and others such as Austria and Germany (especially former Federal Republic of Germany) having much whiter light (Fig. 3 and table S1).

**Fig. 4.**
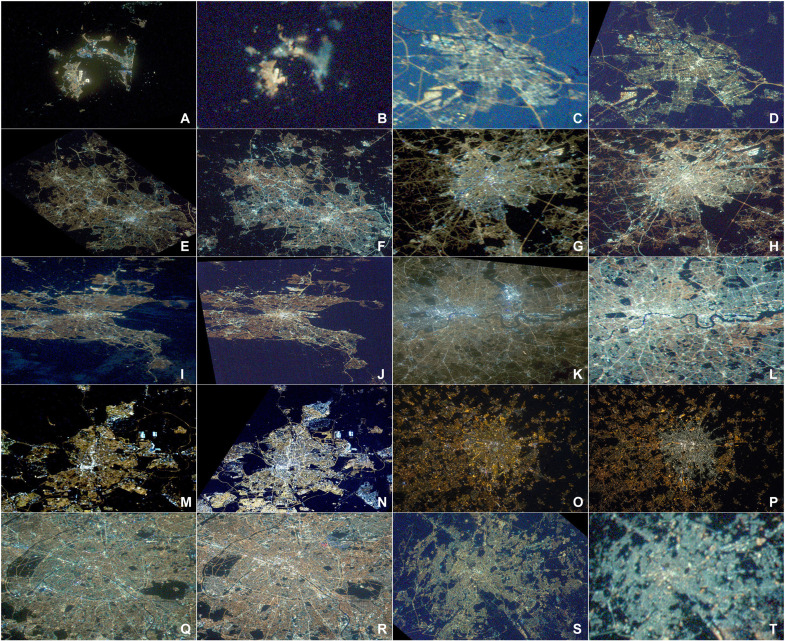
Changes in different European cities at night. Comparison of images obtained from the ISS for Gibraltar/Algeciras in (**A**) 2012 and (**B**) 2020, Amsterdam in (**C**) 2012 and (**D**) 2016, Birmingham in (**E**) 2013 and (**F**) 2020, Brussels in (**G**) 2013 and (**H**) 2019, Dublin in (**I**) 2012 and (**J**) 2014, London in (**K**) 2012 and (**L**) 2020, Madrid in (**M**) 2012 and (**N**) 2017, Milan in (**O**) 2012 and (**P**) 2015, Paris in (**Q**) 2012 and (**R**) 2020, and Rome in (**S**) 2012 and (**T**) 2020. Images (see data file S2) have been color-calibrated following (*49*), except those for Milan, which were processed by the International Astronomical Union.

Across Europe, there were systematic shifts in the frequency distributions of B/G and, particularly, G/R ratios between the two time periods (Fig. 2 and fig. S1), with increases in both B/G [pre-2013 median = 0.35 and interquartile range (IQR) = 0.22, post-2013 median = 0.36 and IQR = 0.17, Kruskal-Wallis χ^2^ = 50.34, df = 1, *P* < 0.001] and G/R values (pre-2013 median = 0.50 and IQR = 0.19, post-2013 median = 0.55 and IQR = 0.17, Kruskal-Wallis χ^2^= 23459.12, df = 1, *P* < 0.001). Synthetic photometry enables these changes to be interpreted in terms of the spectral composition of the light emissions from different kinds of streetlights (*18*). It suggests that in the first period, typical streetlights were predominantly a mixture of high-pressure sodium lamps and white lamps (for example, mercury vapor lights). This is consistent with estimates that in 2005, 56% of the streetlights in Europe were sodium lamps (47% high pressure and 9% low pressure) and 43% were white lights (32% mercury, 3% metal halide, and 8% fluorescent) (*21*). The shift in B/G and G/R ratios by the second period corresponds clearly to the installation of white LED lamps or other white technologies (although LEDs were dominating the market for white light sources) (*22*). These transitions are particularly evident for cities (Fig. 4).

These lighting changes have not been consistent across different countries (table S1). Particularly marked increases in B/G and/or G/R ratios have occurred for Italy, Romania, Ireland, and the United Kingdom. The change of lighting technology took place earlier and more rapidly, for example, across much of Italy; Milan was the first mayoral city in Europe to undertake a total conversion of its street lighting to white LEDs (Fig. 4) (*18*). In the United Kingdom, 51% of all public street lighting was already converted to LEDs by early 2019 (*23*), and in Spain, LEDs were 61% of all sales and 56% of the street lighting by 2017 (*24*). Countries that have experienced less marked changes include Austria and Germany. These countries are traditionally very conservative in their lighting conversions. For example, Germany likely has the highest proportion of gas lighting of anywhere, and a lot of fluorescent and mercury vapor lights are still in use, so the spectral change with LED transition is less marked.

### Changes in light intensity

**Fig. 5.**
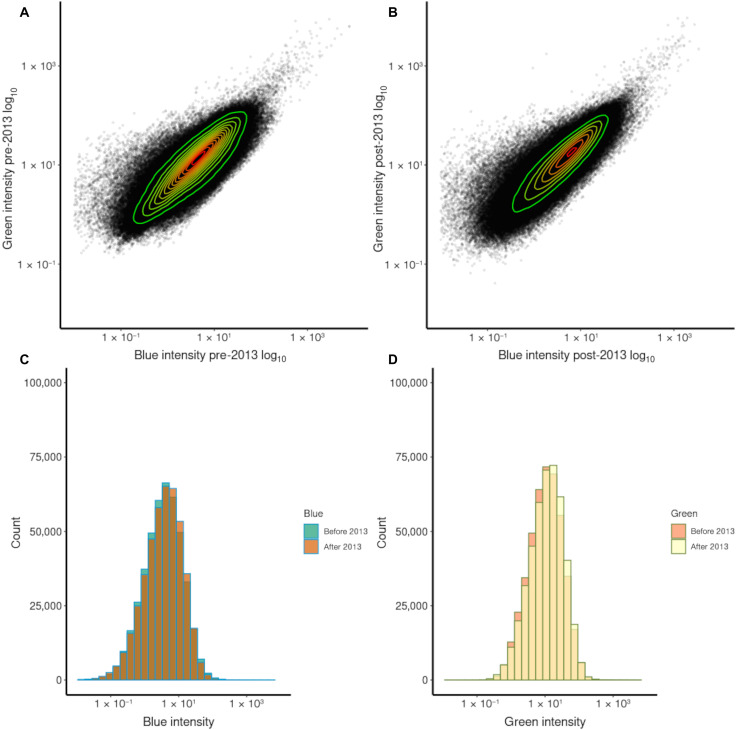
Temporal change in light intensity. Relationships between light intensity values estimated from B and G bands of nighttime light emissions across Europe at 500-m resolution for (**A**) 2012–2013 and (**B**) 2014–2020. Contours show density of points, with red showing the highest density and green showing the lowest density. Histograms of the frequencies of 500-m pixels with different intensity values estimated from (**C**) B and (**D**) G bands for 2012–2013 and 2014–2020.

Previous studies of recent temporal changes in the intensity of ALAN have recognized that these were likely to be underestimated because of their dependence on SNPP/VIIRS-DNB data, which are insensitive to variation in emissions in the blue part of the spectrum (*2*, *18*). We find that between 2012–2013 and 2014–2020 emissions in the G band increased by 11.1%, from a pre-2013 median intensity of 10.59 nW cm^−2^ sr^−1^ (IQR = 17.91) to a post-2013 median of 11.77 nW cm^−2^ sr^−1^ (IQR = 19.66; Kruskal-Wallis χ^2^ = 1366.09, df = 1, *P* < 0.001; Fig. 5), and those in the B band by 24.4%, from a median intensity of 3.6 nW cm^−2^ sr^−1^ (IQR = 6.98) to 3.88 nW cm^−2^ sr^−1^ (IQR = 7.28; Kruskal-Wallis χ^2^ = 166.50, df = 1, *P* < 0.001; Fig. 5). This confirms previous findings that while the LED lighting revolution was promoted as being intended to reduce energy consumption, at national or regional scales, emissions (and likely also energy consumption) have increased (*2*). One potential explanation of this is that conversion to LED street lighting was associated with adoption of a European standard that led to brighter lighting (*16*). Another possibility is the existence of a “rebound effect” or “Jevon’s paradox” in outdoor lighting, where increases in power efficiency and the associated perceived decrease in economic cost have driven increased demand for lighting, and hence, any efficiency gains have been counteracted by increased consumption of light (*25*).

### Biological impacts

The sensitivity of biological traits to different wavelengths of light varies greatly, and spectral response curves have been derived for some key impacts (*13*, *15*). The shifts in spectra of artificial nighttime lighting and especially the increased emissions at blue wavelengths commonly associated with LED street lighting have been found to have substantial biological impacts (*26*–*28*). Here, we focus on how the Europe-wide changes in the spectral composition of emissions have influenced three exemplars, the suppression of melatonin production, the visibility of stars, and the phototaxic response of insects. In addition, we determine how this spectral composition has changed within the geographic ranges of bats, a group with many species that are of conservation concern and for which negative impacts of artificial nighttime lighting, particularly on foraging behavior and movements, have been extensively documented (*29*–*31*).

Melatonin cycles are key components of the circadian systems and determinants of biological temporal organization for a multitude of organisms (*32*, *33*), and the production of this hormone is suppressed by artificial nighttime lighting (*34*). Changes in the melatonin suppression index (MSI) (*35*), estimated from established relationships with B/G and G/R ratios (*18*), reveal that the level of this suppression has increased across Europe between the two periods (using B/G: pre-2013 median = 0.41 and IQR = 0.21, post-2013 median = 0.42 and IQR = 0.21, Kruskal-Wallis χ^2^ = 1253.44, df = 1, *P* < 0.001; using G/R: pre-2013 median = 0.20 and IQR = 0.11, post-2013 median = 0.23 and IQR = 0.13, Kruskal-Wallis χ^2^ = 23459.12, df = 1, *P* < 0.001). Countries that have experienced particularly large increases in these environmental risks include Romania, Spain, and the United Kingdom (table S2).

Increased ALAN emissions, particularly at blue wavelengths (because of the potential for greater atmospheric scattering), have also been predicted to have impacts on the visibility of the stars to some organisms (*1*, *36*). Along with other animals, humans have long used the stars for navigation, but in modern societies, more critical is the concern that loss of views of the natural night sky may have impacts on people’s sense of “nature” and of their place in the universe, as well as impacts on astronomical observations (*1*, *37*). Impacts of changes in ALAN on the visibility of stars can be estimated using the star light index (SLI) on the basis of established relationships between this and B/G and G/R ratios (*18*). As with the MSI, the SLI has increased across Europe between the two time periods, indicating a worsening of the visibility of stars (using B/G: pre-2013 median = 0.64 and IQR = 0.22, post-2013 median = 0.65 and IQR = 0.22, Kruskal-Wallis χ^2^ = 51.45, df = 1, *P* < 0.01; using G/R: pre-2013 median = 0.44 and IQR = 0.14, post-2013 median = 0.47 and IQR = 0.14, Kruskal-Wallis χ^2^ = 23459.12, df = 1, *P* < 0.001).

Increases in emissions at blue wavelengths may also alter the phototaxic response of moths and other insects to artificial nighttime light (*38*). We measured this response using the statistical relationship between the ratio between the phototaxic curve from (*39*) and the G band and G/R ratios, in the same fashion as done for MSI and SLI (*18*). The former ratio has increased across Europe by at least 3% (pre-2013 median = 0.41 and IQR = 0.21, post-2013 median = 0.42 and IQR = 0.21, Kruskal-Wallis χ^2^ = 52.95, df = 1, *P* < 0.001). We also considered as proxy to the total impact not only the spectral composition of the light but also the intensity. We did this by adding the information that we have from SNPP/VIIRS-DNB data and its relationship with the G/R ratio. Considering the intensity on the phototaxic curve, this increased by 10.9% from 3.28 nW cm^−2^ sr^−1^ (IQR = 5.54) to 3.64 nW cm^−2^ sr^−1^ (IQR = 6.12; Kruskal-Wallis χ^2^ = 1253.44, df = 1, *P* < 0.001).

**Fig. 6.**
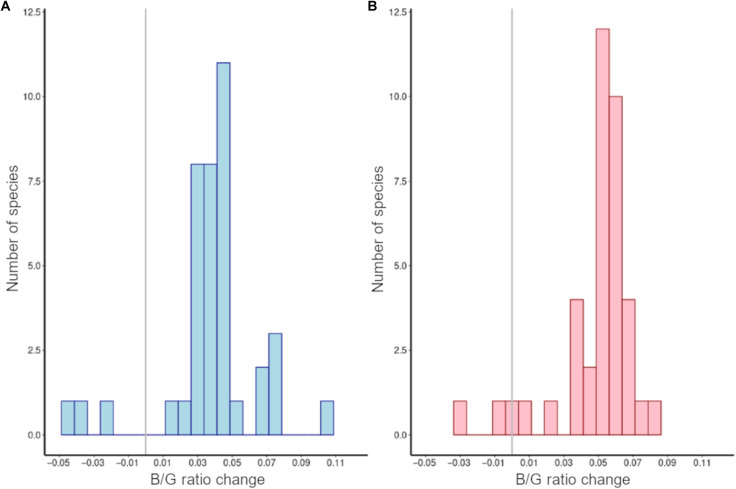
Temporal change in color ratios in the geographic ranges of bat species. Average change in (**A**) B/G ratios and (**B**) G/R ratios of nighttime light emissions at 500-m resolution between 2012–2013 and 2014–2020 within the geographic ranges of bat species in Europe. Vertical lines indicate position of no change.

Last, the spectral composition of nighttime lighting has become whiter, with increases in both B/G and G/R ratios, across the geographic ranges of almost all of the bat species that breed in Europe (Fig. 6). The exceptions are three species, *Plecotus kolombatovici* and *Rhinolophus blasii*, which are restricted to Southeast Europe, and *Myotis alcathoe*, which has a broad distribution not only mainly in France but also some areas of northern Spain and Southeast Europe.

## DISCUSSION

The benefits that LED technology may provide for public lighting, and particularly street lighting, have been much vaunted, with a focus on greater energy efficiency and associated reductions in energy costs and carbon emissions, although the veracity of these claims is typically quite context specific (*16*, *40*); energy use consequences of using LEDs may depend on what technology is being replaced, how the numbers of lamps has changed, the intensity of light emissions, how light emissions change over the operating life of lamps, the source of electricity, and daily timing of demand. There have also been clear opportunities for the use of improved infrastructure (e.g., better positioning and spacing of lamps, better shielding of lamp emissions, and centralized lighting management systems) during the retrofitting of public lighting with LED lamps. However, the increased use of LEDs in public lighting has also led to intentional shifts in the spectral composition of the associated emissions, with these often becoming whiter, with the intention of providing improved color rendering for human vision. Here, using the unique opportunity provided by the DSLR images taken by astronauts aboard the ISS, we have shown that across the past decade, these spectral changes have been widespread across Europe. They have been spatially very uneven, reflecting historical variation in the kinds of lighting systems that have been used and the diversity of national and regional lighting policies and approaches, but the net effect has nonetheless been a pronounced whitening of the artificial light that is eroding natural nighttime cycles across the continent.

We have demonstrated that one consequence of this shift in the spectral composition of artificial nighttime lighting emissions has been a marked progressive underestimation in the intensity of those emissions as determined by the principal satellite platform used for this purpose (SNPP/VIIRS-DNB). This is a simple outcome of the spectral sensitivity of the associated sensors. However, this may have very important implications because the emission intensities measured by SNPP/VIIRS-DNB are used for a wide diversity of purposes, including the estimation of levels of human population density and urbanization (*41*, *42*), economic activity (*43*) and wilderness areas (*44*), and the impacts of the coronavirus disease 2019 pandemic (*45*, *46*).

Last, we show that another consequence of the recent increased whitening of artificial nighttime lighting emissions across much of Europe has been an increased likelihood of negative biological impacts of that lighting. We have focused on estimating these impacts on a few key responses (melatonin suppression, visibility of starlight, phototaxis, and behavior of bats). However, these impacts will be much more extensive than these examples, given that very many biological phenomena are spectrally dependent on and often particularly sensitive to blue emissions (*34*, *36*).

The benefits of LED technology do not rest principally in the ability to produce broad white light emissions (this is also achievable with other technologies). Nonetheless, attempts to gain these other benefits while reducing the biological impacts using LEDs (in new developments or retrofitting schemes) to implement lighting systems without strong emissions at shorter wavelengths have, to date, been limited (*47*). Rather, the present trajectory of change is anticipated to continue. It will be important to monitor these changes. While systematic, high-resolution multispectral nighttime satellite imagery remains the ideal (and proposals and initial attempts to produce this have been made) (*48*–*50*), in the absence of such data at present composite color [red-green-blue (RGB)], images from the ISS represent a unique resource for monitoring and mapping environmental risks due to ALAN.

## MATERIALS AND METHODS

### Images

Following extensive searches, images of areas of Europe taken by astronauts aboard the ISS between 2012–2013 and 2014–2020 (the different durations reflect the availability of images) were downloaded from the NASA Johnson Space Center (*51*). These were obtained using Nikon D3, D3S, D4, and D5 DSLR cameras. Images are courtesy of the Earth Science and Remote Sensing Unit, NASA Johnson Space Center. Astronauts and cosmonauts from NASA, the European Space Agency, the Canadian Space Agency–Agence Spatiale Canadienne, the Japan Aerospace Exploration Agency, and ROSCOSMOS were involved in their acquisition. The full list of images used in the mosaicked maps is given in data file S1.

### Processing

Selected (focal) images were passed individually through a pipeline of calibration and correction, as detailed in (*52*), with the following steps: Decodification - the raw file for a focal image is transformed into standard “fits” or “tiff” files, and the individual RGB channels are separated. Linearity correction - the nonlinear regime of the camera at high photon counts is corrected. Flat field/vignetting - the nonhomogeneous illumination of the sensor by the camera lens is corrected. Spectral characterization of the channels - the difference in spectral sensitivity of each channel of the camera compared with the others is corrected. Astrometric calibration - reference stars, measurements from which are used for absolute calibration of the focal images, are located on calibration images. Georeferencing - ground control points are located within the focal images to transform them onto georeferenced coordinates using thin-plate splines in GDAL (Geospatial Data Abstraction Library) (*53*). Photometric calibration (stars) – in the calibration images, the absolute, already known, flux of the stars is determined to ascertain the instrumental constant that allows photon counts on the focal images to be converted into SI units. Radiometric correction (settings correction: by exposure time, ISO, lens transmittance, etc.) - instrumental constant calculated using the stars is applied to the focal images and corrected by the settings of the camera used in image acquisition. Atmospheric correction - remaining factors, including aerosols and Raleigh scattering, are corrected for. The occurrence of clouds in focal images was masked manually, by visual inspection of the JPG images and removing blurred areas and any areas that appeared dark when compared with SNPP/VIIRS-DNB data.

Calibrated and corrected images were spatially mosaicked to create separate maps for those obtained during 2012–2013 and during 2014–2020. Where images overlapped for any given area centers of images were used preferentially (these having better signal-to-noise ratios because of the vignetting effect toward the edges). Similarly, images obtained at low observation angles (i.e., closer to the nadir) were preferred because of the effects of atmospheric absorption and the curvature of Earth on signal-to-noise ratios and those images obtained with longer focal length lenses. For the post-2013 map, more recent images were preferred to make this as up to date as possible. To reduce noise in the final maps, outliers on the B/G versus G/R diagram [see (*18*)] were removed if ratios exceeded values of 1.2 for B/G or G/R and if R/G exceeded 6. This procedure is applied because of the different signal-to-noise ratios of the different images of the mosaic. They were also masked using SNPP/VIIRS-DNB imagery from the Earth Observation Group (EOG) of NOAA/EOG Colorado School of Mines version (to match most closely the dates on which the ISS images were obtained) (*54*) at a threshold intensity of 0.5 nW cm^−2^ sr^−1^ to avoid calibration issues due to natural skyglow (*55*).

Last, the two mosaicked maps were clipped to the boundary of Europe, including Albania, Austria, Belgium, Bosnia and Herzegovina, Bulgaria, Croatia, Czech Republic, France, Germany, Greece, Hungary, Ireland, Italy, Lithuania, Luxembourg, Montenegro, The Netherlands, Poland, Portugal, Romania, Serbia, Slovakia, Slovenia, Spain, Switzerland, and the United Kingdom. Data for additional countries were not of adequate quality (e.g., images only available at high observation angles) or were not available for inclusion.

### Changes in light intensity

To characterize absolute brightness of light emissions, the changes in intensity were determined in the G and B bands for the 2012–2013 and 2014–2020 maps. Because of the uncertainty as to which window of the ISS the images have been acquired from and the effects of the tilt of the acquisition, the absolute intensity values for the G and B bands have larger uncertainty than do the band ratios, as all bands would be affected equally according to the transmittance values known for the ISS windows. A more accurate way, therefore, to estimate changes in the G and B bands is using the intensity measured from VIIRS (an average of several images) corrected by the G/R and B/G ratios, respectively. We use the statistical relationship between the G/R ratio and G/VIIRS and that between the B/G ratio and B/VIIRS (fig. S2) for the most common street lighting spectra using the same synthetic photometry techniques as described in (*18*).

This way, we define the G light intensity as

$$G_{\text{VIIRS}}=\text{VIIRS}∙\frac{G_{\text{ISS}}}{\text{VIIRS}}[G_{\text{ISS}}/R_{\text{ISS}}]$$ (1)

where

$$\frac{G_{\text{ISS}}}{\text{VIIRS}}=0.21_{-0.06}^{+0.21}∙1.5_{-0.30}^{+0.09}G_{\text{ISS}}/R_{\text{ISS}}$$ (2)

Superscripts and subscripts are 95% confidence intervals on the parameter values as determined by bootstrapping.

In a similar way, we calculate the B light intensity but using the B/G ratio instead of the G/R ratio

$$B_{\text{VIIRS}}=\text{VIIRS}∙\frac{B_{\text{ISS}}}{\text{VIIRS}}[B_{\text{ISS}}/G_{\text{ISS}}]$$ (3)

where

$$\frac{B_{\text{ISS}}}{\text{VIIRS}}=-0.03_{-0.04}^{+0.03}+0.6_{-0.20}^{+0.20}∙B_{\text{ISS}}/G_{\text{ISS}}+1.3_{-0.30}^{+0.20}∙{\left[B_{\text{ISS}}/G_{\text{ISS}}\right]}^{2}$$ (4)

### Biological impacts

The change in both B/G and G/R ratios of artificial nighttime light emissions between 2012–2013 and 2014–2020 drives biological responses. In addition, the influence of changes in the spectral composition was determined specifically for (i) melatonin suppression, using relationships established between the MSI and the B/G and G/R ratios (*18*); (ii) visibility of stars, using relationships established between the SLI and the B/G and G/R ratios (*18*); and (iii) the relative phototaxic response of moths and total phototaxic response of moths. In the case of the phototaxic response, we used a similar procedure as above (*18*), where

$$P_{I}=\text{VIIRS}∙\frac{P}{\text{VIIRS}}[G_{\text{ISS}}/R_{\text{ISS}}]$$ (5)

where

$$\frac{P}{\text{VIIRS}}=0.23_{-0.03}^{+0.05}∙0.21_{-0.21}^{+0.06}G_{\text{ISS}}/R_{\text{ISS}}$$ (6)

We also determined how B/G and G/R ratios changed between 2012–2013 and 2014–2020 within the geographic ranges of European bat species. We used the species list from (*56*). Range data for 38 species were downloaded from (*57*); no range data were available for *Barbastella caspica*, *Eptesicus anatolicus*, and *Miniopterus pallidus*. The range data were cropped to the Europe mask and reprojected to ETRS89-extended/LAEA Europe, EPSG: 3035, an equal area projection for Europe.

### Analyses of change

When analyzing changes in spectra, light intensity, and biological impacts, we focus on the Europe-wide shifts, rather than changes in individual pixels. This avoids assuming perfect co-registration of pixels between the two time periods, given the challenges of georeferencing images obtained at quite different angles to the nadir. The Root Mean Squared Error (RMSE) for georeferencing error is estimated at approximately 4 pixels [see (*52*)].
