# Supplementary material for: Environmental risks from artificial nighttime lighting widespread and increasing across Europe
Source: Sci Adv. 2022 Sep 14;8(37):eabl6891. doi: 10.1126/sciadv.abl6891 (PMC9473566; doi:10.1126/sciadv.abl6891)
Supplement: Supplementary file 1 — Figs. S1 to S3 Tables S1 and S2 [file sciadv.abl6891_sm.pdf]

Science Advances
AAAS

# Supplementary Materials for

## Environmental risks from artificial nighttime lighting widespread and increasing across Europe

Alejandro Sánchez de Miguel *et al.*

Corresponding author: Kevin J. Gaston, k.j.gaston@exeter.ac.uk

*Sci. Adv.* **8**, eabl6891 (2022)
DOI: 10.1126/sciadv.abl6891

**The PDF file includes:**

Figs. S1 to S3
Tables S1 and S2

**Other Supplementary Material for this manuscript includes the following:**

Data S1 and S2

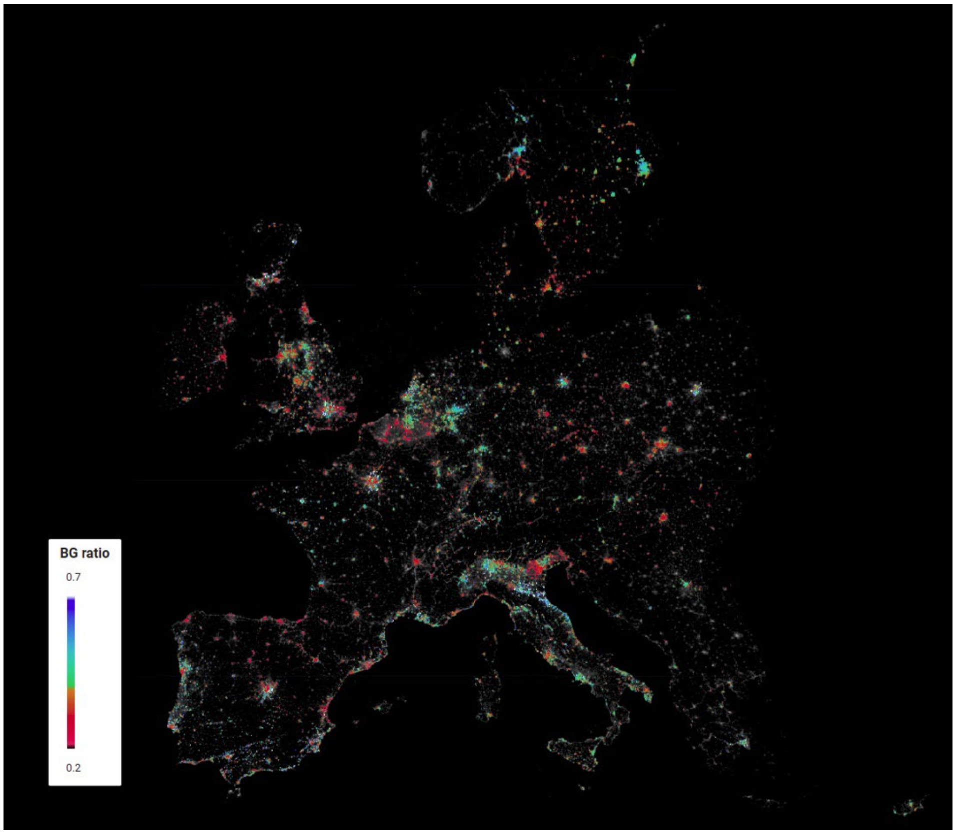


(a)

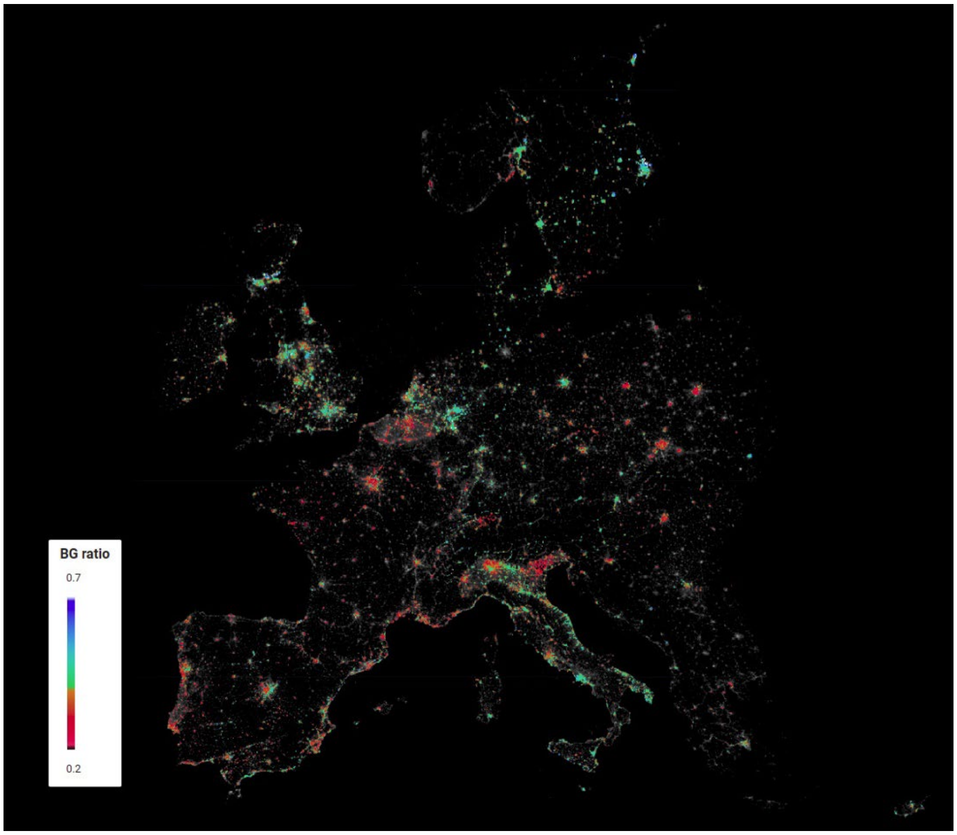


(b)

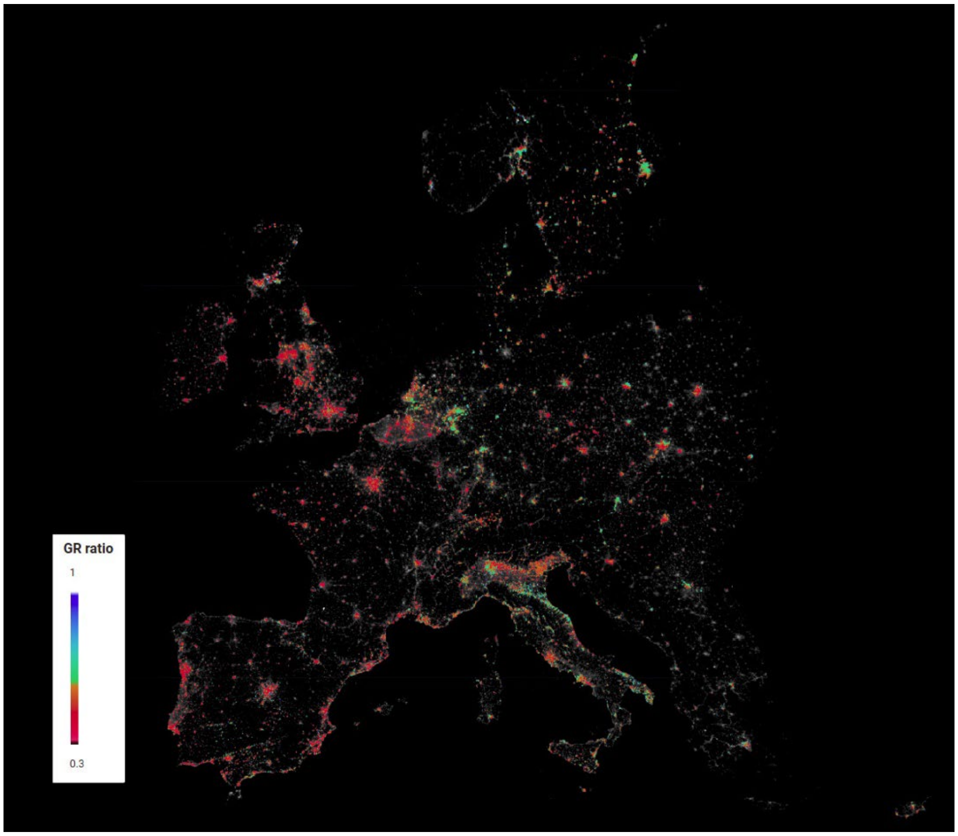


(c)

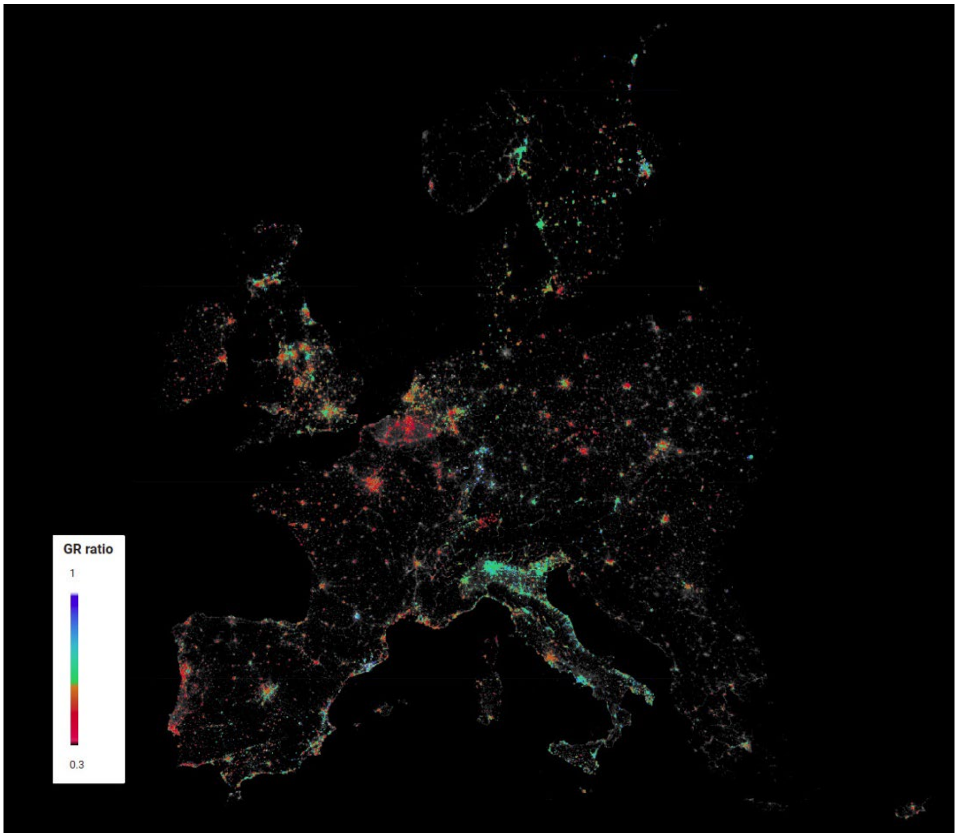


(d)

**Fig. S1.** Variation in (a) B/G pre-2013, (b) B/G post-2013, (c) G/R pre-2013 and (d) G/R post-2013.

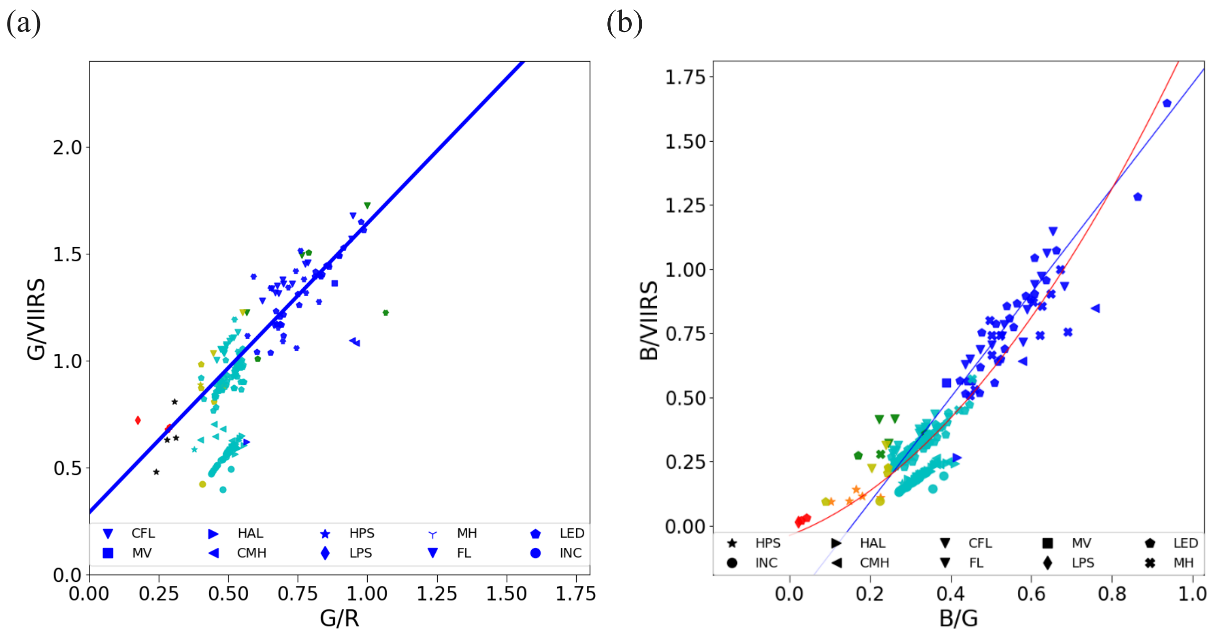


**Fig. S2**. Relationships between the ratios of (a) G and (b) B bands with VIIRS intensity and the (a) G/R and (b) B/G band ratios determined for the spectra of different kinds of lamps (different symbol shapes; HAL - Halogen, MH - Metal Halide, CMH - Ceramic Metal Halide, CFL - Compact Fluorescent, FL - Fluorescent, HPS - High Pressure Sodium, LPS - Low Pressure Sodium, and INC – Incandescent). Symbol colours represent different broad classes of blue content (see *18*). See (*18*) for further details.

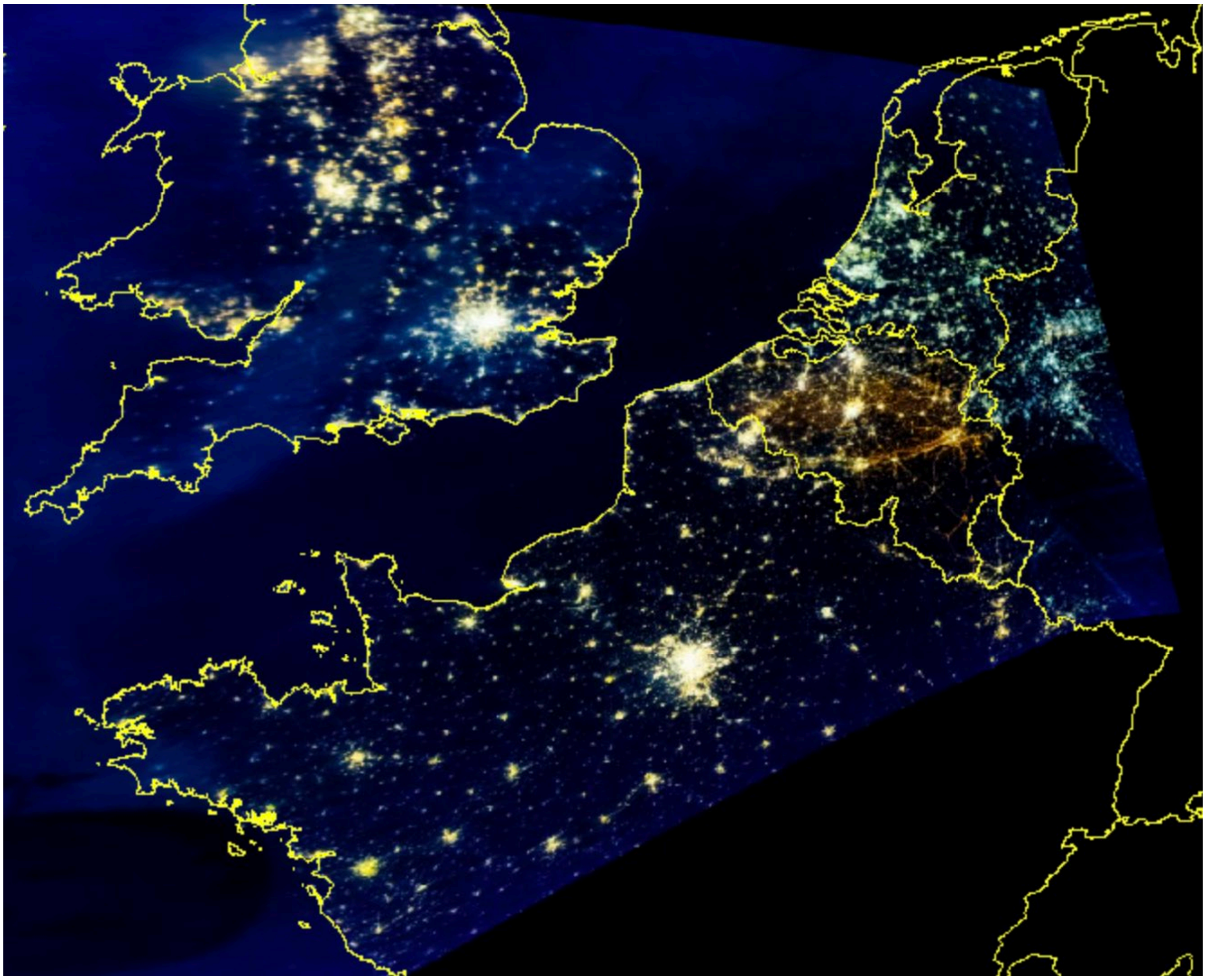

**Fig. S3**. Reprojected version of Fig. 3 with country boundaries.

**Table S1.** Changes in colour ratios of nighttime light emissions for different European countries. For each country the median and I.Q.R. of blue/green (B/G) and green/red (G/R) ratios are given for 2012-2013 and 2014-2020, the Kruskal-Wallis Chi-squared values testing whether the ratio values (per pixel) for the two time periods originate from the same distribution, and the associated significance levels.

| Country | B/G ratio | | | | G/R ratio | | | |
|---|---|---|---|---|---|---|---|---|
| | pre-2013 | post-2013 | K-W | p-value | pre-2013 | post-2013 | K-W | p-value |
| Albania | 0.38± 0.18 | 0.41± 0.12 | 14.11 | <0.001 | 0.53± 0.23 | 0.63± 0.28 | 57.87 | <0.001 |
| Austria | 0.41± 0.10 | 0.41± 0.17 | 1.42 | 0.23 | 0.60± 0.11 | 0.65± 0.23 | 337.15 | <0.001 |
| Belgium | 0.3± 0.16 | 0.31± 0.15 | 2.91 | 0.09 | 0.45± 0.15 | 0.44± 0.14 | 146.86 | <0.001 |
| Bosnia & Herzegovina | 0.49± 0.15 | 0.51± 0.11 | 43.04 | <0.001 | 0.61± 0.17 | 0.67± 0.22 | 40.04 | <0.001 |
| Bulgaria | 0.33± 0.11 | 0.47± 0.12 | 283.61 | <0.001 | 0.54± 0.26 | 0.61± 0.24 | 59.89 | <0.001 |
| Croatia | 0.33± 0.09 | 0.33± 0.12 | 4.80 | <0.03 | 0.48± 0.11 | 0.5± 0.17 | 79.74 | <0.001 |
| Czech Rep. | 0.31± 0.10 | 0.32± 0.14 | 137.27 | <0.001 | 0.45± 0.12 | 0.47± 0.16 | 219.48 | <0.001 |
| France | 0.34± 0.16 | 0.29± 0.15 | 4400.39 | <0.001 | 0.45± 0.16 | 0.49± 0.17 | 2011.91 | <0.001 |
| Germany | 0.38± 0.14 | 0.40± 0.18 | 94.10 | <0.001 | 0.55± 0.17 | 0.56± 0.21 | 292.37 | <0.001 |
| Greece | 0.37± 0.20 | 0.32± 0.17 | 191.68 | <0.001 | 0.55± 0.24 | 0.53± 0.19 | 75.10 | <0.001 |
| Hungary | 0.31± 0.07 | 0.32± 0.11 | 14.59 | <0.001 | 0.47± 0.10 | 0.52± 0.16 | 848.95 | <0.001 |
| Ireland | 0.28± 0.13 | 0.35± 0.17 | 947.69 | <0.001 | 0.39± 0.13 | 0.49± 0.17 | 2036.93 | <0.001 |
| Italy | 0.38± 0.21 | 0.38± 0.16 | 249.75 | <0.001 | 0.55± 0.20 | 0.64± 0.23 | 11867.79 | <0.001 |
| Lithuania | 0.35± 0.04 | 0.40± 0.076 | 109.61 | <0.001 | 0.43± 0.25 | 0.51± 0.11 | 37.14 | <0.001 |
| Luxembourg | 0.35± 0.09 | 0.32± 0.15 | 31.36 | <0.001 | 0.39± 0.14 | 0.49± 0.18 | 303.04 | <0.001 |
| Montenegro | 0.33± 0.06 | 0.51± 0.11 | 173.07 | <0.001 | 0.47± 0.07 | 0.52± 0.31 | 7.23 | <0.01 |
| Netherlands | 0.38± 0.16 | 0.38± 0.15 | 0.03 | 0.86 | 0.53± 0.17 | 0.54± 0.16 | 16.45 | <0.001 |
| Poland | 0.34± 0.09 | 0.31± 0.13 | 824.59 | <0.001 | 0.49± 0.16 | 0.52± 0.17 | 408.62 | <0.001 |
| Portugal | 0.38± 0.24 | 0.32± 0.11 | 2530.39 | <0.001 | 0.44± 0.16 | 0.47± 0.16 | 352.27 | <0.001 |
| Romania | 0.30± 0.11 | 0.42± 0.21 | 741.24 | <0.001 | 0.54± 0.17 | 0.63± 0.29 | 112.03 | <0.001 |
| Serbia | 0.41± 0.12 | 0.37± 0.092 | 255.50 | <0.001 | 0.57± 0.13 | 0.55± 0.17 | 35.45 | <0.001 |
| Slovakia | 0.36± 0.10 | 0.37± 0.17 | 0.32 | 0.57 | 0.48± 0.11 | 0.55± 0.22 | 214.62 | <0.001 |
| Slovenia | 0.37± 0.09 | 0.39± 0.098 | 49.54 | <0.001 | 0.55± 0.11 | 0.61± 0.21 | 227.63 | <0.001 |
| Spain | 0.30± 0.19 | 0.34± 0.17 | 1080.73 | <0.001 | 0.48± 0.22 | 0.54± 0.25 | 2677.89 | <0.001 |
| Switzerland | 0.31± 0.21 | 0.31± 0.17 | 1.61 | 0.20 | 0.48± 0.20 | 0.51± 0.2 | 174.88 | <0.001 |
| UK | 0.33± 0.2 | 0.39± 0.16 | 3532.42 | <0.001 | 0.46± 0.16 | 0.54± 0.2 | 9290.65 | <0.001 |

**Table S2**. Changes in Melatonin Suppression Index (MSI) for different European countries. For each country the median and I.Q.R. MSI estimated based on blue/green (B/G) and green/red (G/R) ratios are given for 2012-2013 and 2014-2020, the Kruskal-Wallis Chi-squared values testing whether the ratio values (per pixel) for the two time periods originate from the same distribution, and the associated significance levels.

| **Country** | **MSI B/G** | | | | **MSI G/R** | | | |
|---|---|---|---|---|---|---|---|---|
| | **pre-2013** | **post-2013** | **K-W** | **p-value** | **pre-2013** | **post-2013** | **K-W** | **p-value** |
| Albania | 0.44± 0.22 | 0.48± 0.15 | 14.11 | <0.001 | 0.22± 0.13 | 0.28± 0.16 | 57.87 | <0.001 |
| Austria | 0.48± 0.12 | 0.48± 0.2 | 1.14 | 0.28 | 0.26± 0.06 | 0.29± 0.13 | 337.15 | <0.001 |
| Belgium | 0.34± 0.19 | 0.35± 0.19 | 3.11 | 0.08 | 0.18± 0.09 | 0.17± 0.08 | 146.86 | <0.001 |
| Bosnia & Herzegovina | 0.58± 0.19 | 0.61± 0.14 | 43.04 | <0.001 | 0.27± 0.10 | 0.30± 0.13 | 40.04 | <0.001 |
| Bulgaria | 0.39± 0.13 | 0.56± 0.15 | 283.61 | <0.001 | 0.22± 0.15 | 0.27± 0.14 | 59.89 | <0.001 |
| Croatia | 0.39± 0.11 | 0.38± 0.15 | 4.67 | 0.03 | 0.19± 0.06 | 0.20± 0.10 | 79.75 | <0.001 |
| Czech Rep. | 0.35± 0.12 | 0.37± 0.18 | 137.27 | <0.001 | 0.17± 0.07 | 0.19± 0.10 | 219.48 | <0.001 |
| France | 0.39± 0.20 | 0.34± 0.19 | 4137.33 | <0.001 | 0.17± 0.09 | 0.2± 0.097 | 2011.91 | <0.001 |
| Germany | 0.45± 0.18 | 0.47± 0.23 | 95.90 | <0.001 | 0.23± 0.10 | 0.24± 0.12 | 292.37 | <0.001 |
| Greece | 0.43± 0.25 | 0.37± 0.21 | 194.45 | <0.001 | 0.23± 0.14 | 0.22± 0.11 | 75.10 | <0.001 |
| Hungary | 0.36± 0.09 | 0.38± 0.13 | 14.59 | <0.001 | 0.19± 0.05 | 0.22± 0.09 | 848.95 | <0.001 |
| Ireland | 0.31± 0.16 | 0.41± 0.21 | 943.64 | <0.001 | 0.14± 0.07 | 0.20± 0.10 | 2036.93 | <0.001 |
| Italy | 0.45± 0.26 | 0.45± 0.19 | 233.38 | <0.001 | 0.23± 0.12 | 0.28± 0.13 | 11867.79 | <0.001 |
| Lithuania | 0.41± 0.05 | 0.47± 0.09 | 109.61 | <0.001 | 0.16± 0.15 | 0.21± 0.06 | 37.14 | <0.001 |
| Luxembourg | 0.41± 0.11 | 0.38± 0.18 | 31.36 | <0.001 | 0.14± 0.08 | 0.20± 0.11 | 303.04 | <0.001 |
| Montenegro | 0.39± 0.08 | 0.60± 0.13 | 173.07 | <0.001 | 0.19± 0.04 | 0.21± 0.18 | 7.23 | 0.01 |
| Netherlands | 0.44± 0.20 | 0.45± 0.18 | 0.01 | 0.92 | 0.22± 0.10 | 0.23± 0.09 | 16.45 | <0.001 |
| Poland | 0.39± 0.11 | 0.36± 0.17 | 804.71 | <0.001 | 0.20± 0.09 | 0.21± 0.10 | 408.62 | <0.001 |
| Portugal | 0.44± 0.30 | 0.36± 0.14 | 2571.09 | <0.001 | 0.17± 0.09 | 0.18± 0.09 | 352.27 | <0.001 |
| Romania | 0.35± 0.14 | 0.49± 0.26 | 741.24 | <0.001 | 0.23± 0.10 | 0.28± 0.17 | 112.03 | <0.001 |
| Serbia | 0.48± 0.15 | 0.43± 0.11 | 255.50 | <0.001 | 0.25± 0.07 | 0.23± 0.10 | 35.45 | <0.001 |
| Slovakia | 0.42± 0.13 | 0.43± 0.21 | 0.32 | 0.57 | 0.19± 0.07 | 0.23± 0.13 | 214.62 | <0.001 |
| Slovenia | 0.43± 0.11 | 0.45± 0.12 | 49.54 | <0.001 | 0.23± 0.06 | 0.26± 0.12 | 227.63 | <0.001 |
| Spain | 0.35± 0.24 | 0.39± 0.20 | 1090.03 | <0.001 | 0.19± 0.13 | 0.23± 0.14 | 2677.89 | <0.001 |
| Switzerland | 0.36± 0.26 | 0.36± 0.21 | 2.78 | 0.10 | 0.19± 0.12 | 0.21± 0.12 | 174.88 | <0.001 |
| UK | 0.38± 0.25 | 0.46± 0.20 | 3466.50 | <0.001 | 0.18± 0.09 | 0.23± 0.12 | 9290.65 | <0.001 |

**Data S1** List of ISS images used to form final mosaiced maps.

**Data S2 List of ISS images used in Fig. 4.**
